# Comparing subsampling strategies for efficient pairwise analysis of large pathogen genomic and spatial datasets: An application to *Mycobacterium tuberculosis* transmission

**DOI:** 10.1371/journal.pcbi.1014353

**Published:** 2026-09-23

**Authors:** Yu Lan, Chieh-Yin Wu, Hsien-Ho Lin, Ted Cohen, Joshua L. Warren

**Affiliations:** 1 Department of Epidemiology of Microbial Diseases, Yale School of Public Health, New Haven, Connecticut, United States of America; 2 College of Public Health, National Taiwan University, Taipei, Taiwan; 3 Department of Biostatistics, Yale School of Public Health, New Haven, Connecticut, United States of America; The University of Melbourne Faculty of Science, AUSTRALIA

## Abstract

Pairwise analysis of genomic and spatial data offers opportunities to identify and estimate the associations between covariates and the transmission of pathogens between individuals. However, such pairwise analyses are computationally intensive, and may not be feasible to conduct given the high dyad count in even moderately sized datasets. Here we compare two approaches to increase the efficiency of pairwise analysis for large datasets. We quantify and compare the performance of divide-and-conquer Bayesian model fitting and pairwise case-control approaches for estimating associations between individual- and pair-level covariates and shared membership in a transmission cluster. We utilize a large dataset (n = 4,154) of spatially-referenced, genomically-sequenced *Mycobacterium tuberculosis* isolates collected from a single city for this analysis, and conduct simulation studies under three representative tuberculosis genomic clustering settings. Across all simulation studies, the case-control approach produced negligible bias and expected 95% credible interval coverage, and performed comparably to the divide-and-conquer approach, with a somewhat narrower distribution of bias estimates and fewer outliers when effect sizes were large. Thus, we recommend using the case-control approach with five controls per case to downscale datasets for pairwise analysis when analysis of the entire dataset is not possible. This approach mitigates the computational challenges of pairwise Bayesian modeling on datasets that require significant computational resources while maintaining desired inferential properties.

## Author summary

Pairwise analyses of large datasets to study pathogen transmission are computationally demanding because they typically require simultaneous analysis of each possible pair of individuals in a dataset; as datasets become larger these analyses often are not feasible to conduct even with access to high-performance computing resources. In this work, we compare a case-control approach and divide-and-conquer approaches for more efficient pairwise analysis of large datasets. Using a large dataset of *Mycobacterium tuberculosis* isolates including genetic and spatial data, we investigate the performance of each method for estimating the associations between host covariates and genetic clustering of isolates. We find that the case-control approach is generally preferred over methods which first divide the data into subsets and then combine results. While additional extensions of these analyses are needed to test the generality of these findings to other data settings, this work provides a practical way forward for the pairwise analysis of large datasets to study pathogen transmission.

## 1. Introduction

Tuberculosis (TB) is caused by *Mycobacterium tuberculosis* (Mtb), a bacterium transmitted from person to person through the respiratory route. Whole genome sequencing (WGS) can be used to identify possible instances of person-to-person Mtb transmission based on the genetic relatedness of sequenced isolates [1]. Many Mtb studies have applied a threshold for the maximum number of genetic differences (i.e., single nucleotide polymorphisms or SNPs) between isolates to identify possible transmission linkages [2], and other specialized approaches (e.g., TransPhylo [3]; Outbreaker [4]) estimate the probability of transmission between individuals based on genetic distances as well as other factors related to the accumulation of genetic differences between cases (e.g., timing of diagnoses, bacterial mutation rates). With the increased availability of high resolution genomic and spatial data, a growing number of studies have also combined spatial data with genomic relatedness to study Mtb transmission in communities [5].

Network-based regression modeling of spatially-referenced pairwise genetic similarity measures offers a novel opportunity to understand which factors are associated with Mtb transmission, while accounting for multiple sources of correlation (e.g., network dependence and spatial correlation) [6]. These models are designed to associate pairwise measures of genetic similarity (e.g., SNP distances or shared cluster membership) between two individuals with individual- and pair-specific covariates of interest. However, as the number of individuals in a study increases, the number of unique pairs increases dramatically, leading to extreme computational demands. For a dataset with n > 1 individuals, there are n(n-1)/2 dyadic observations when directionality of transmission is not considered; this results in computational challenges particularly when working in the Bayesian setting.

Employment of subsampling strategies with high-performance computing (HPC) resources is one possible approach to address this computational challenge. One promising sampling strategy is the case-control approach [7,8]. Furthermore, some studies have discussed different sampling designs, such as a two-step case-control sampling scheme for studying binary outcomes [9], optimal sampling for large sample logistic regression [10], and sampling with replacement and Poisson sampling in optimal subsampling [11].

Another computational strategy is the divide-and-conquer approach, where the large dataset is divided into subsets, analysis is conducted on each subset, and the posterior results are combined across all analyses [12]. Several methods have been developed based on this strategy to handle the analysis of large geostatistical datasets, such as spatial meta-kriging for point data [13] and scalable Bayesian modelling on areal (lattice) count data [14]. Regardless of types of data, methods have been developed to approximate posteriors samples from analyses simultaneously run on subsets of the entire dataset. The Consensus Monte Carlo method [15] approximates the complete posterior distribution by aggregating weighted averages of subposterior samples from independent Markov chain Monte Carlo (MCMC) runs. Alternatively, the semiparametric density product estimator method (DPE) estimates the subposterior density using kernel density estimation and thus combines estimated densities of subposteriors to approximate the full posterior density [16]. Despite extensive discussions in the existing literature on Bayesian inference for transmission networks for infectious diseases [17] and epidemiology more broadly [18,19], few studies have focused on the applications of these approaches to dyadic data.

In Section 2, we introduce and conduct a pairwise analysis on three large (n = 700) benchmark datasets with different pairwise clustering proportions from the full dataset collected in Kaohsiung, Taiwan. These three datasets were near the maximum size that we could analyze given our computational resources. We perform a pairwise analysis on these datasets to obtain reference estimates which we use to benchmark and compare estimates derived from competing methods described in Section 3. Section 3 describes the simulation study which we use to compare competing methods for obtaining estimates from subsampled datasets. Based on the results from the simulation study, we apply the top performing methods to the entire Mtb dataset from Kaohsiung (n = 4,154) and report the results in Section 4. We conclude with recommendations for pairwise analysis using Bayesian models for large dyadic datasets in Section 5.

## 2. Benchmark data and application

### 2.1. Benchmark data

We use a population-based dataset of 4,154 individuals with culture-confirmed TB in Kaohsiung from 2019 to 2023, with linked data on genomic cluster, age, and geolocation of residence for each individual. Additional details of the study from which these individuals were identified have been previously published [20]. The size for our benchmark subsamples (i.e., n = 700) is close to the upper limit of pairwise analysis that we can process given our available computational resources. We perform this pairwise analysis on a partition of Bouchet HPC cluster at the Yale Center for Research Computing. Jobs were executed on CPU nodes equipped with Intel Xeon Gold 6426Y processors. Each job used 1 CPU core and 200 GB of allocated memory that supports jobs up to seven days in duration.

In this study, we define genomic clusters using a 12 SNP threshold. We assign pairs of individuals belonging to the same genomic cluster as having a binary shared cluster membership outcome equal to 1; all other pairs are assigned an outcome of 0. To capture varying proportions of genetic clustering from recent TB literatures (see S1 Table and S1 Text in the support information), we construct three benchmark datasets (see Fig 1) characterized by different pairwise clustering proportions, defined as the fraction of pairs where the binary outcome is equal to 1 among the total number of unique pairs (hereafter referred to simply as pairs). Each dataset consists of 700 isolates with clustering proportions of 0.008, 0.002, and 0.0009, selected from the full dataset (n = 4,154). We note that the clustering proportion of the full dataset is 0.0006. Although clustering proportions were varied substantially across studies (as shown in S1 Fig), we consider these three values to represent plausible and meaningfully different clustering scenarios observed in the tuberculosis genomic epidemiology literature. Although those values are small in absolute terms, a clustering proportion of 0.008 is four times higher than 0.002, which is itself more than twice 0.0009. Even a clustering rate of 0.002 represents a relatively high level of clustering in the context of TB.

**Fig 1:**
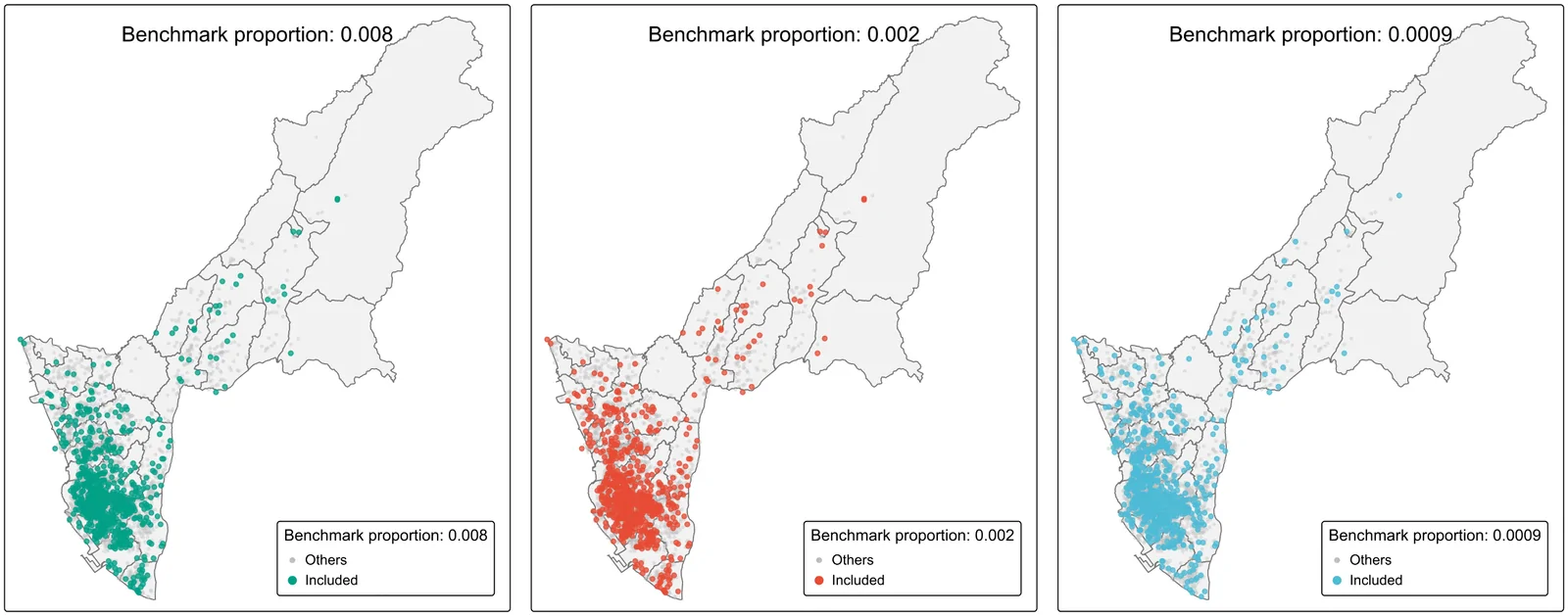
Spatial distribution of individuals with Mtb in Kaohsiung, Taiwan (2019–2023), jittered for privacy. The colored points show three sets of 700 isolates sampled from the full dataset to create the benchmark estimates with pairwise clustering proportions of 0.008, 0.002, and 0.0009. The Kaohsiung boundary data were obtained from Taiwan’s Government Open Data platform (https://data.gov.tw/en/datasets/7441).

To construct each benchmark dataset, we retained all cases from selected genomic clusters to preserve the transmission structure within the included clusters. The remaining cases were then randomly sampled from the rest of the population to achieve the target pairwise clustering proportion for each benchmark dataset. To obtain a dataset with a relatively high clustering proportion (0.008), we include all isolates from small and medium-sized clusters (2 < the number of isolates<20, n = 563) and randomly selected an additional 167 isolates; to obtain a dataset with a median clustering proportion (0.002), we include all isolates from small and medium-sized clusters (<5 isolates, n = 606) and randomly selected an additional 94 isolates; to obtain a dataset with a low clustering proportion (0.0009), we include all isolates from small clusters (<3 isolates; n = 346) and randomly selected an additional 354 isolates.

These sets of 700 individuals comprise 244,650 pairs; 1,930, 487, and 213 of these dyads are linked within a Mtb genomic cluster (see Table 1). We consider only the unique pairs among individuals given that our goal is to determine whether they are infected with very similar isolates of Mtb (and thus potentially members of the same transmission chain), rather than to infer the direction of transmission.

**Table 1:** Genomic cluster sizes and counts of pairs for three benchmark datasets.

Clustering proportion	Cluster size	Numbers of clusters	Total unique pairs
**0.008**	3	52	156
	4	27	162
	5	9	90
	6, 7	3	45, 63
	8, 9, 10, 13, 14, 18	2	56,72,90,156,182,306
	11, 12, 15, 17, 20	1	55,66,105,136,190
	**Total**	**94**	**1,930**
**0.002**	2	175	175
	3	52	156
	4	26	156
	**Total**	**253**	**487**
**0.0009**	2	181	181
	3	2	5
	4	2	12
	6	1	15
	**Total**	**186**	**213**

### 2.2. Pairwise analysis

We first use the *GenePair* R package [6] to conduct a pairwise analysis on the benchmark datasets. The primary analysis uses a hierarchical Bayesian logistic regression framework that accounts for network dependence and spatial correlation to assess the association between pairwise and individual-level risk factors and transmission. The model included three pairwise covariates: the combined age of the two individuals, the absolute age difference of the two individuals, and the spatial distance between the two individuals’ residential locations.

The logistic regression model is given as


$$\begin{array}{c} {\;Y}_{ij}|p_{ij}\sim \text{Bernoulli}\left(p_{ij}\right),\;i=\text{1},\ldots ,m-\text{1},\;j=i+\text{1},\ldots ,m, \end{array}$$ (1)

$$\begin{array}{c} \text{ logit}\left(p_{ij}\right)=\mu +{𝐱}_{ij}^{\text{T}}\beta +(d_{i}+d_{j})\gamma +\eta_{i}+\eta_{j} \end{array}$$

where $Y_{ij}$ is the binary outcome describing if individuals *i* and *j* are in the same genomic cluster (1) or not (0); ${𝐱}_{ij}$ is a vector of the covariates describing the difference (i.e., the age difference and the spatial distance); ${(d}_{i}+d_{j})$ represents the covariate specific for individual i and individual j (i.e., the combined age); m is the number of individuals; $\eta_{i}$ is the individual-specific random effect parameter modeled as a function of separable independent and spatially-correlated components, with the independent component modeled using a Gaussian distribution with mean equal to zero and unknown variance, and the spatial component modeled using a Gaussian process with exponential spatial correlation structure. The random effect for a single individual is observed across multiple observations (i.e., the same individual is represented across multiple dyads, each involving a different partner and geographic distance). This within-individual variation in distance improves identifiability of both the random effects and the association between geographic distance and the probability of being genetically clustered. Full details are given in Warren et al. [6]. The model is fitted in the Bayesian setting using MCMC sampling techniques. For each parameter, we computed posterior means and 95% highest posterior density credible intervals on the odds ratio scale.

### 2.3. Results

Analysis of the benchmark datasets reveals that smaller age difference, a younger combined age, and shorter spatial distance between the two individuals with Mtb were independently associated with being members of the same genomic cluster (see Fig 2). We treat these parameter estimates as the reference value for comparing the performances of the different competing computational methods.

**Fig 2:**
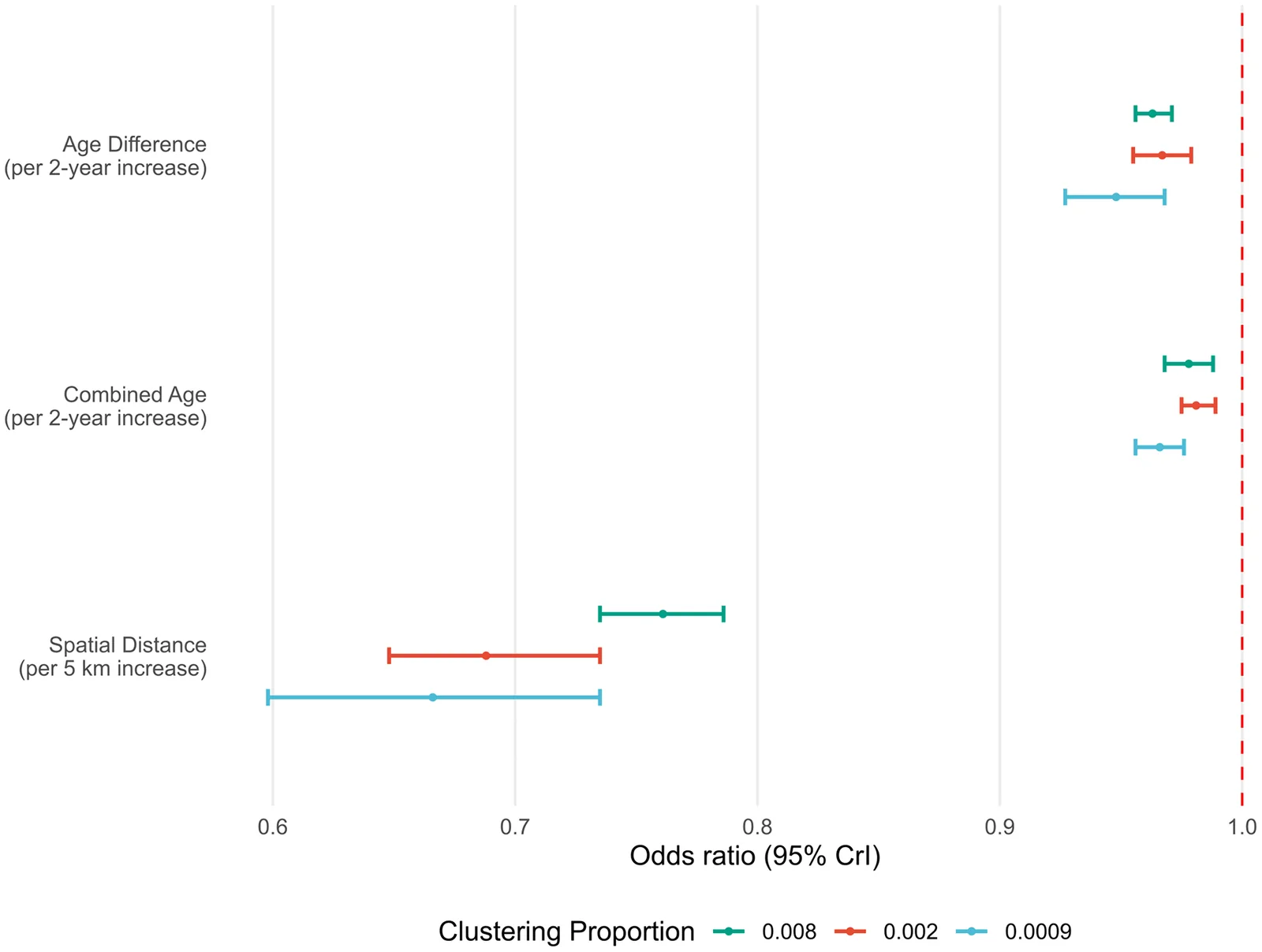
Odds ratio posterior median and 95% credible intervals for predictors in benchmark datasets at different clustering proportion.

## 3. Comparative analysis using simulated data

### 3.1. Data generation

We simulate data from the model in (1) to evaluate the performance of the case-control and the divide-and-conquer approaches, measured by the bias of point estimates and coverage of credible intervals. The data generation process is illustrated in Fig 3. We first estimate parameters based on the GenePair results from three benchmark datasets with 700 individuals. Then, we randomly select 700 new individuals from the full dataset of 4,154 individuals for simulation purposes. Using new individuals for each simulated dataset ensures that our results are more generalizable to the full Kaohsiung dataset.

**Fig 3:**
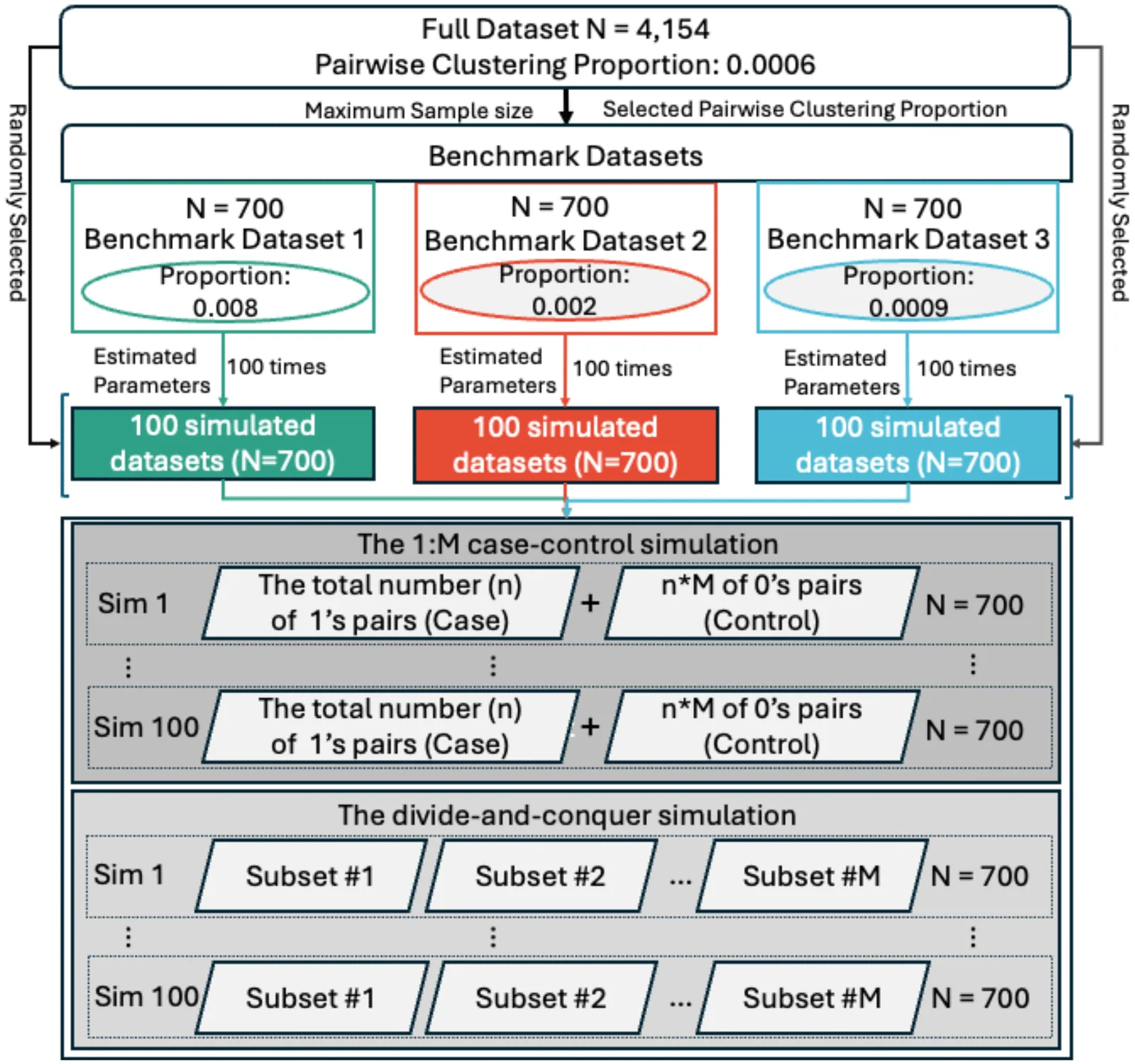
Data generation process for simulated datasets.

For each pair of isolates, we use the posterior median parameter estimates from the benchmark analyses to simulate the binary outcome ($Y_{ij}$) (i.e., whether two isolates from per the subset belonged to the same genomic cluster) using the observed covariate values for the individuals. For each simulated dataset (n = 700), we generate a new realization of the individual-specific random effect parameters to avoid conditioning on a single set that ultimately define the level of correlation in the data. To do so, we use posterior median estimates of the correlation parameters to simulate new values from both the non-spatial and spatial components for each dataset. For each clustering proportion, we simulate 100 datasets for analysis. We note that the clustering proportion of each simulated dataset is not fixed, as different individuals are randomly selected in each simulation. Nevertheless, the resulting clustering proportion is close to the target clustering proportion specified in the simulation design.

### 3.2. Competing methods

We applied the case-control and the divide-and-conquer approaches on the same simulated dataset of different clustering proportion and compared their results.

We investigate the case-control strategy as follows: we first include all case pairs from the simulated dataset (1 as outcome), randomly select M_control_ times as many pairs as the number of case pairs for control pairs (0 as outcome), and combine both selected pairs, including their individual and pairwise information, to conduct pairwise analysis. No matching or other relationship between case pairs and the sampled control pairs was required. In other words, a control pair could consist of any two individuals that did not belong to the same genomic cluster, including: (1) two individuals from different genomic clusters, (2) two individuals who did not belong to any genomic cluster, or (3) one individual from a genomic cluster and one individual who did not belong to any cluster. This sampling strategy ensured that the control pairs represented the overall population of non-clustered pairs and did not preferentially favor any specific pairing type. We compare the results when increasing the number of randomly selected controls (M_control_ = 1, 3, 5, and 10) per case to evaluate the impact of the number of controls selected per case. We repeat the control selection procedure for each of the four values of M_control_ on the 100 simulated datasets and run GenePair on each simulated dataset.

We investigate the divide-and-conquer strategy as follows: we divide the dataset into subsets, conduct the pairwise analysis on each subset, and combine posterior results using different statistical methods across subsets. To evaluate the impact of subset number (and therefore subset size), we conduct GenePair analysis on three values of subsets (M_subset_ = 3, 5, and 10). We repeat the subsampling procedure for each of the three values of M_subset_ on the 100 simulated datasets and ran GenePair on each simulated dataset. We use six combination methods to combine the posterior results from subsets.

We denote Z=(Y, 𝐱) as the full dataset of responses and predictors. For the Bayesian pairwise analysis, the marginal posterior distribution for the regression parameters is denoted as $\rho (\beta^{*}|Z)$, where $\beta^{*}\;$is the vector of regression parameters corresponding to the previously mentioned covariates (i.e., combined age, age difference, and spatial distance).

For the divide-and-conquer strategy, we divide the dataset into $M_{subset}$ non-overlapping subsets $z_{m}$, $m=\text{1},\;\ldots M_{subset}.$ We denote $\beta^{*}$ as the d-dimensional parameter vector of interest, and ${\left\{{\beta^{*}}_{m}^{s}\right\}}_{s=\text{1}}^{S}$ as the s posterior samples of the parameter vector $\beta^{*}$ obtained from subset m, where $s=\text{1},\;\ldots S\text{ and }m=\text{1},\;\ldots M_{subset}$ (after ensuring convergence). We evaluate different methods that combine the posterior samples ${\left\{{\beta^{*}}_{m}^{s}\right\}}_{s=\text{1}}^{S}$ across subsets to approximate samples from the full dataset posterior distribution $\rho \left(\beta^{*}|Z\right)$. We conduct GenePair analysis on each subset and obtain MCMC samples from the posterior distribution of parameters. The six divide-and-conquer methods we include are:

- Meta-analysis (Meta) using a fixed-effect model: The final point estimate ${{\overset{―}{\beta }}^{*}}_{meta}$ is computed as ${{\overset{―}{\beta }}^{*}}_{meta}=\;\frac{\sum_{m=\text{1}}^{M}w_{m}{{\overset{―}{\beta }}^{*}}_{m}}{\sum_{m=\text{1}}^{M}w_{n}}$, where ${{\overset{―}{\beta }}^{*}}_{m}=\frac{\text{1}}{S}\sum_{s=\text{1}}^{S}{\beta^{*}}_{m}^{s},$ is the posterior mean estimate of the vector for subset *m*, and$\;w_{m}$ is a vector of the inverse of the sample variances for each regression parameter for subset *m*.
- Sample average (sampleAvg): Approximate samples from the full data posterior distribution of $\beta^{*}$ by averaging the posterior samples from all subsets at each sample s (i.e., ${\beta^{*}}_{\text{avg}}^{s}=\frac{\text{1}}{M}\sum_{m=\text{1}}^{M}{\beta^{*}}_{m}^{s}$).
- The Consensus Monte Carlo method: Combines independent posterior samples across subsets into pooled posterior samples. For each MCMC iteration s, the combined posterior samples is given by: ${\beta^{*}}_{\text{CMC}}^{s}={\left(\sum_{m=\text{1}}^{M}W_{m}\right)}^{-\text{1}}(\sum_{m=\text{1}}^{M}W_{m}{\beta^{*}}_{m}^{s})$, where ${\beta^{*}}_{m}^{s}$ = $({\beta^{*}}_{m\text{1}}^{s},\;{\beta^{*}}_{m\text{2}}^{s}$,…, ${\beta^{*}}_{md}^{s}{)}^{\text{T}}$ is the s-th posterior sample of $\beta^{*}$ from subset m, and $W_{m}$ denotes the inverse of the covariance matrix from subset *m*. The definition of the weight matrix $W_{m}$ depends on the assumed structure of $\beta^{*}$:
- MCindep: Assumes independence among model parameters $\beta^{*}$. Thus, $W_{m}$ is a diagonal matrix, where each diagonal entry is the inverse of the sample variance of covariate $\beta_{d}^{*}$, and${\;W}_{mj}$ is the inverse of the variance of the marginal posterior samples collected for parameter *j* after analyzing subset *m*.
- MCcov: Assumes covariance among model parameters $\beta^{*}$. In this case, $W_{m}$ is defined as the inverse of the sample variance-covariance matrix estimated from subset m.
- DPE: Unlike other methods that directly combine posterior samples, DPE does not produce combined posterior samples directly. The full-data posterior is approximated by: ${\hat{\rho }}_{DPE}\left(\beta^{*}|Z\right)\propto \;\prod_{m=\text{1}}^{M}{\hat{\rho }}_{m}(\beta^{*}|Z)$, where ${\hat{\rho }}_{m}(\beta^{*}|Z)$ denotes the estimated subposterior density for subset m, obtained as the product of a parametric density and a nonparametric correction estimated via kernel smoothing. We calculate the bandwidth matrix used in kernel density estimation based on Silverman’s rule of thumb [21]. We include two bandwidth parameters in our analysis:
- DPE1 employes the original bandwidth matrix.
- DPE2 scales the original bandwidth matrix by a factor of 2 to apply greater smoothing.

We apply Meta using the *metafor* package in R [22]. We apply the other MCMC posterior combined methods (i.e., sampleAvg, MCindep, MCcov, DPE1, DPE2) using R *parallelMCMCcombine* package [23].

For each strategy, we assess bias (i.e., the difference between the estimated median per simulation and the reference value), the proportion of simulation replicates in which the CrI contains the reference value, and the average 95% CrI width for each covariate individually, including the combined age, the age difference, and spatial distance.

All simulations were performed on the previously described HPC cluster. Jobs were executed managed using the SLURM workload manager, with each job (i.e., one case-control simulation or one subset from the divide-and-conquer simulation) allocated 1 CPU core and 20 GB of memory.

### 3.3. Simulation results and comparison

For both the case-control and divide-and-conquer analyses, we collected 10,000 posterior samples after discarding the first 10,000 iterations as burn-in and thinning the remaining iterations by a factor of 5 to reduce autocorrelation. For the divide-and-conquer analysis, this procedure was applied separately to each subset, after which the subset-specific posterior samples were combined to obtain the final results. All MCMC chains exhibited satisfactory convergence across all simulation scenarios. Although slightly slower mixing was observed for the lower clustering proportions when using 10 subsets, the chains remained stable and showed no evidence of non-convergence (see representative trace plots in S2–S4 Figs).

Fig 4 shows the results for the case-control strategy using different controls per case based on all 100 simulations. Bias is shown as boxplots of the simulation-based bias estimates, together with the mean bias and its 95% confidence interval (Fig 4A). Coverage is shown as the estimated coverage rate with its 95% confidence interval (Fig 4B). Overall, the case-control strategy performs well with low bias and expected 95% CrI coverage for all regression parameters. For each of the three clustering proportions tested, the method with one control per case performed worse than when we increased the numbers of controls. Across each clustering proportion, increasing the number of controls per case led to narrower 95% credible intervals for all three factors, indicating improved precision (see S5 Fig).

**Fig 4:**
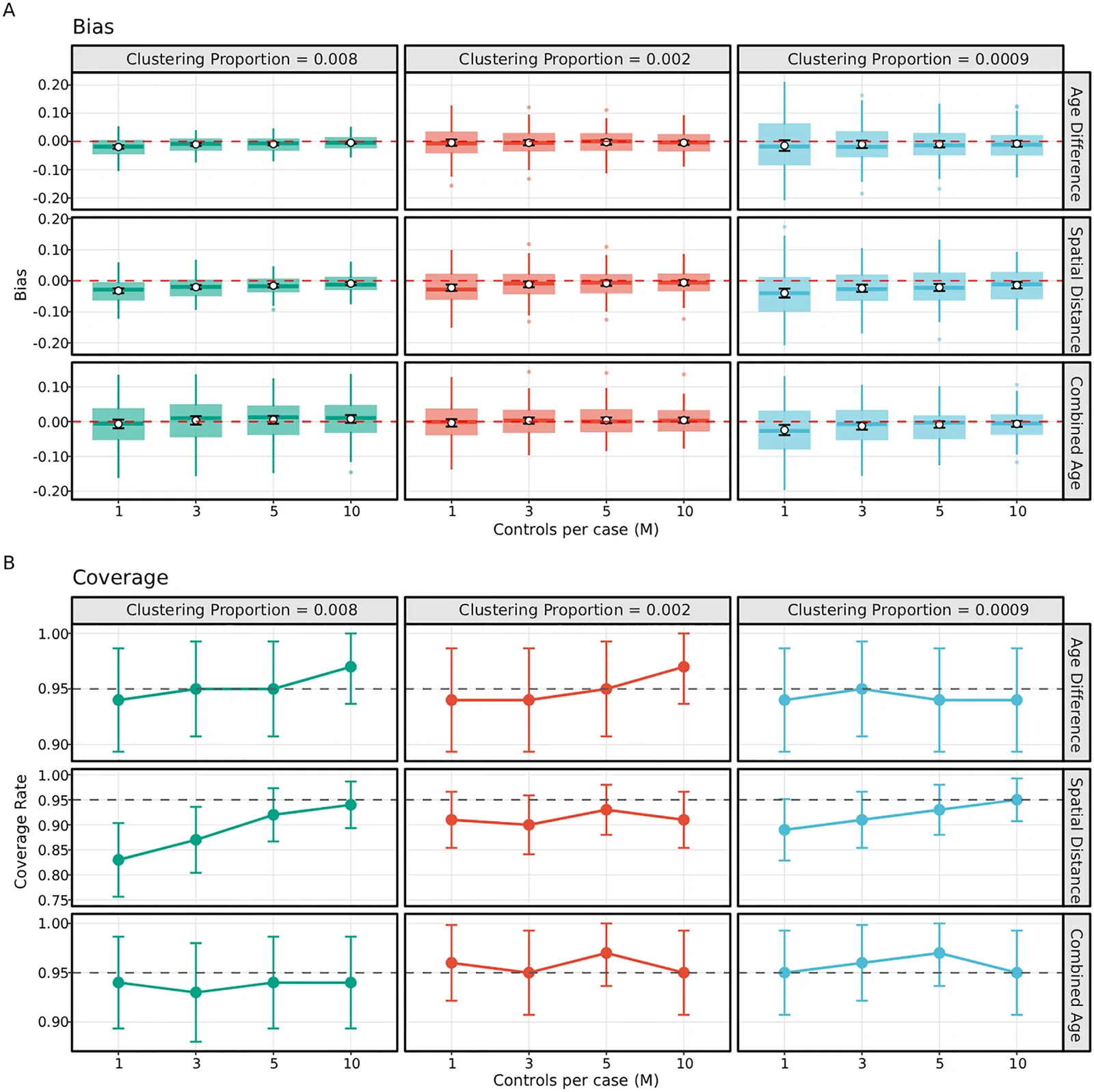
Estimated bias and empirical 95% credible interval coverage simulation study results by factors and methods using the case-control approach. Error bars show 95% confidence intervals for the estimated bias and coverage.

Fig 5 shows the results for each method using the divide-and-conquer strategy, excluding the sampleAvg method as this method has larger bias and wider CrIs than the other methods tested (see S6 Fig). Overall, each method with three subsets performs best, and Meta was consistently among the methods that performed best with small bias and high coverage across three covariates and different types of partitions. For each clustering proportion, the divide-and-conquer approach shows wider 95% credible intervals as the number of subsets increases, indicating reduced precision as we increase the number of partitions (see S7 Fig).

**Fig 5:**
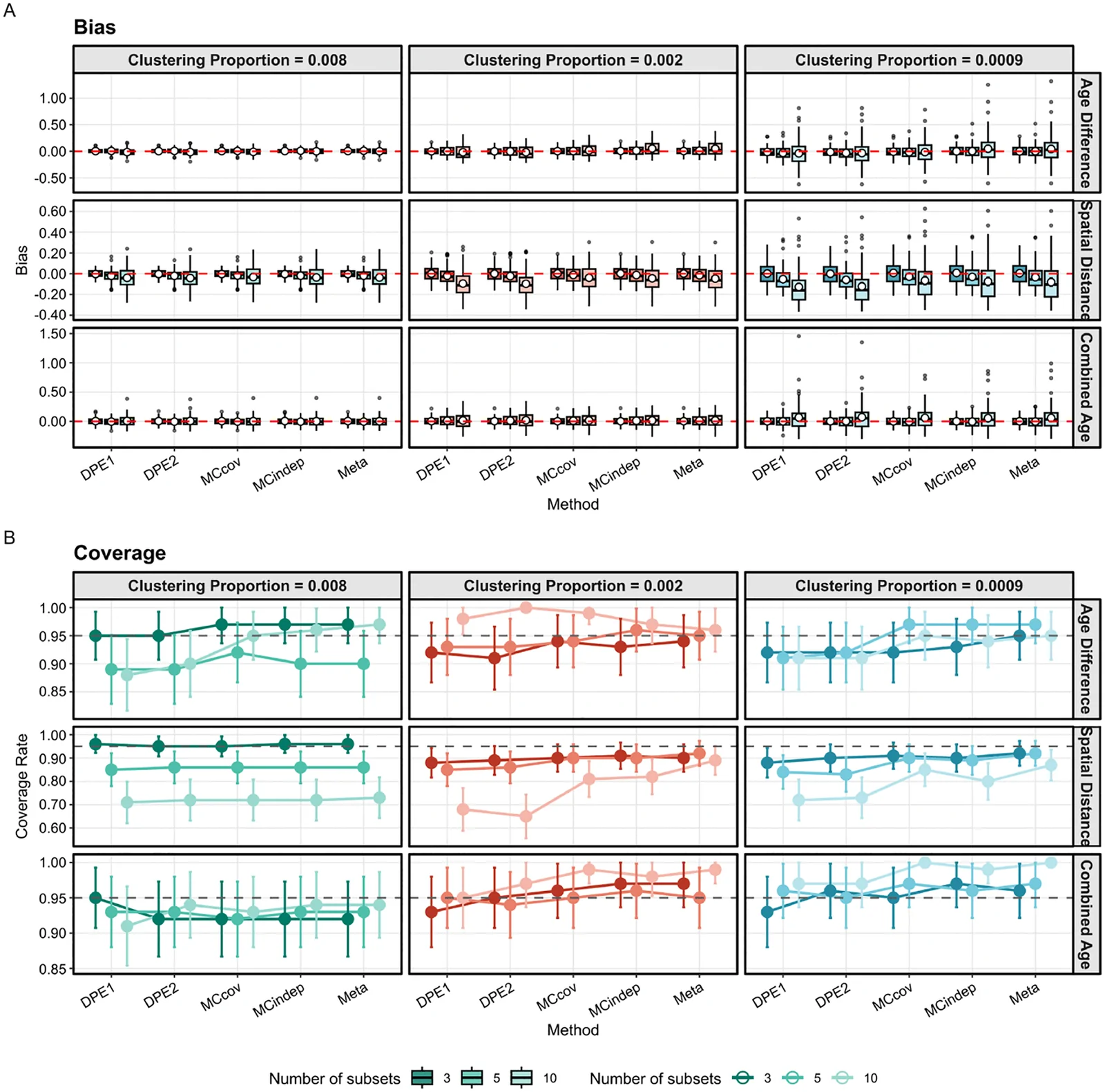
Estimated bias and empirical 95% credible interval coverage simulation study results by factors and methods using the divide-and-conquer approach. Error bars show 95% confidence intervals for the estimated bias and coverage.

Notably, in our analyses the spatial distance covariate consistently has greater bias and lower coverage compared with the non-spatial factors. When we divide the full dataset into ten subsets, the coverage rate of DPE methods decreases to approximately 70% for this variable. As shown in Fig 2, the spatial covariate is associated with the largest effect size, suggesting that this issue may not be unique to the spatial covariate, but may instead be most apparent for covariates with large effect sizes. To test this hypothesis, we swapped the effect sizes of age difference and spatial distance in the simulated datasets and observed that the poorer performance shifted to age difference (see S8 Fig). This suggests that the relatively poor performance under the divide-and-conquer approach is driven by large effect sizes under sparse clustered pair information, rather than being a result of the spatial nature of this covariate.

In Fig 6, we compare the two best performing methods (i.e., lowest bias and highest coverage) from each strategy: the case-control approach with five controls per case and the divide-and-conquer approach with three subsets using the Meta method to combine subposteriors. Both methods show mean bias close to zero and similar distribution for the bias, although the divide-and-conquer method shows a wider range and a greater number of outliers. Both methods show expected coverage for the two age-related factors, while the case-control approach shows a better coverage rate for the spatial distance factor than the divide-and-conquer method. We also compare credible interval lengths of each factor estimated by the two approaches; the case–control method produces consistently narrower intervals than the divide-and-conquer method, suggesting greater precision (i.e., less posterior uncertainty) (see S9 Fig).

**Fig 6:**
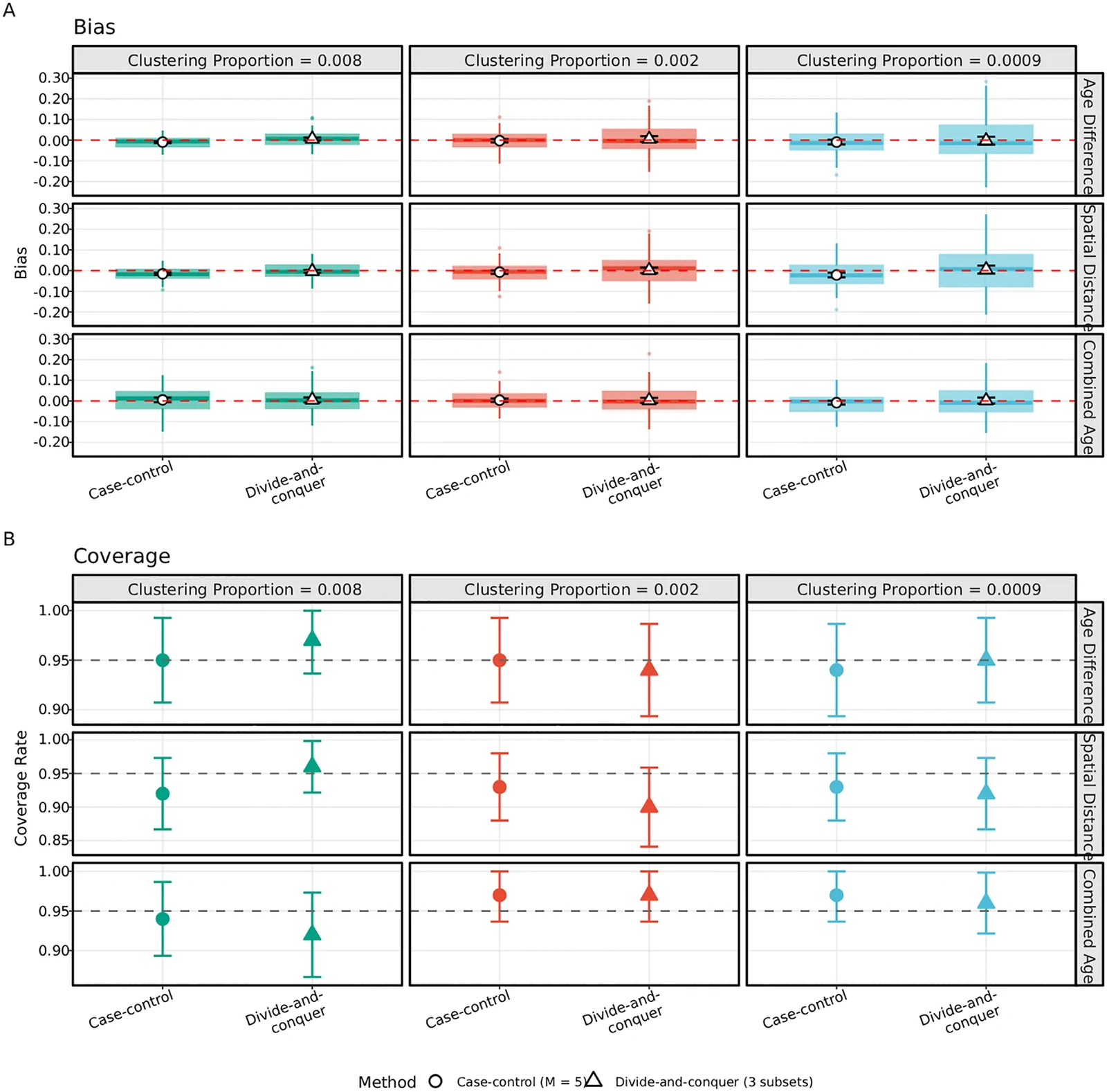
Performance comparison between the case-control approach with five control per case and the divide-and-conquer approach with three subsets using the Meta method to combine. Estimated bias and empirical 95% credible interval coverage simulation study results by factors and methods. Error bars show 95% confidence intervals for the estimated bias and coverage.

For the full-data analysis of benchmark datasets with 60,000 posterior samples, the longest runtime was observed at a clustering proportion of 0.008 (4,907.6 minutes), followed by 0.0009 (4,311.9 minutes) and 0.002 (4,201.7 minutes). This suggests that the higher clustering proportion contributes to a longer runtime, whereas differences between the two lower clustering proportions are minimal and may be difficult to distinguish due to variability in the HPC computing environment. In general, both subsampling strategies show a marked improvement in terms of the total computation time as shown in Fig 7. The longest average computation time was observed for the case-control approach using 10 controls per case on the simulated dataset with a clustering proportion of 0.008, representing a more than five-fold reduction.

**Fig 7:**
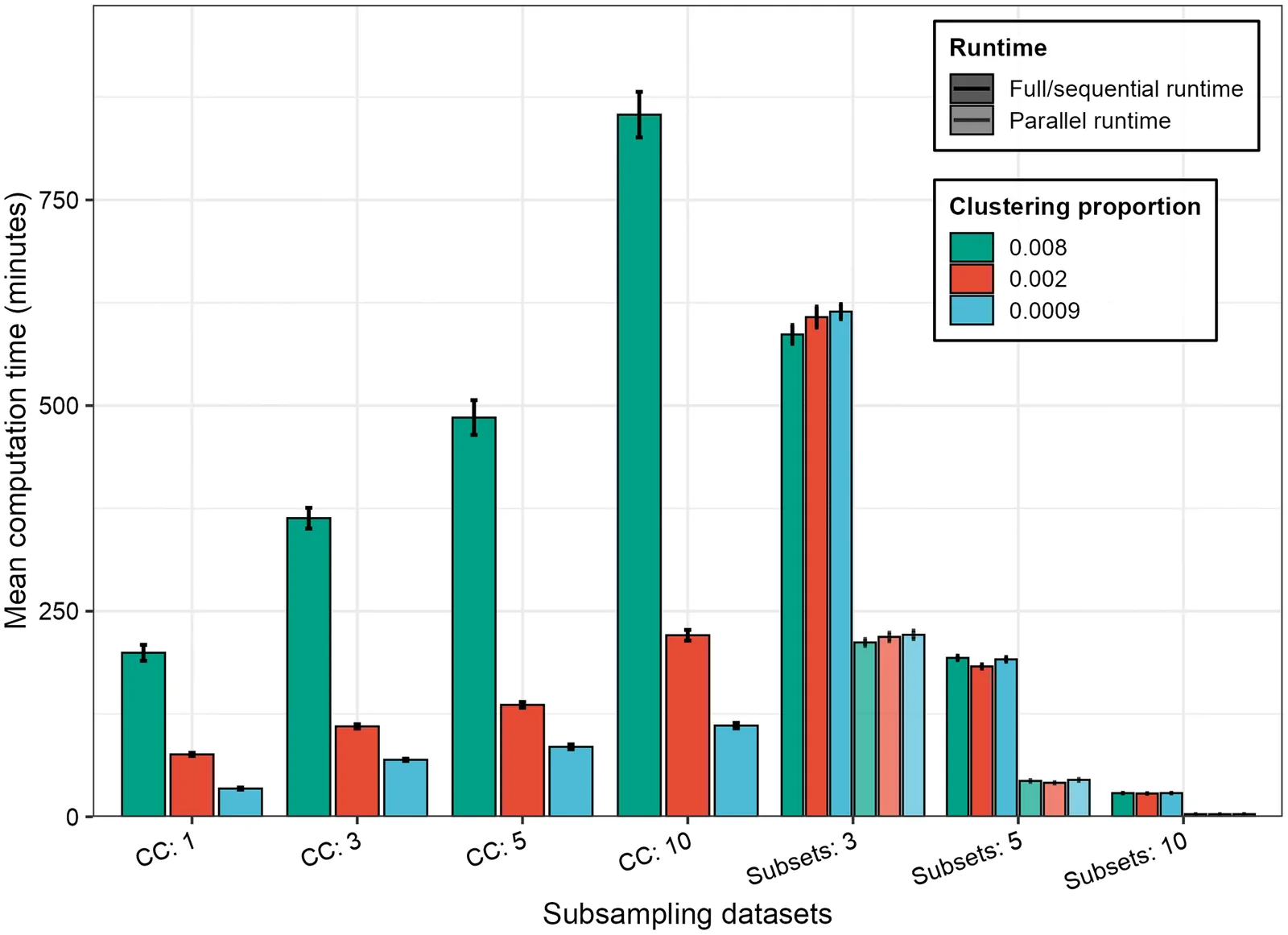
Computation time by subsampling dataset and methods, with average runtime labeled (in minutes). Colored bars indicate different clustering proportions. CC denotes the case-control approach; for example, CC:1 indicates that one control was selected per case. For the divide-and-conquer approach, dark-colored bars indicate computation time measured as the sum of runtime across all subdivisions, and light-colored bars indicate average runtime from parallel execution (i.e., all subsets ran parallel).

Fig 7 compares computing time for the different methods. For the divide-and-conquer strategy, the reported computation time includes only the pairwise analyses and does not include the time required to combine the results from the subsets. The results further show that datasets with a larger clustering proportion require a longer runtime when using the case-control strategy. This relationship is not observed for the divide-and-conquer strategy. In other words, computation time for the case-control approach appears to be driven by the number of 1’s pairs, while computation time for the divide-and-conquer approach mainly depends on the subsample size in each subdivision. The divide-and-conquer strategy also achieved a substantially shorter parallel runtime.

Based on the results from two strategies, we conclude that the case-control approach generally outperforms the divide-and-conquer approach. For those three pairwise clustering proportions, using five controls per case seems to provide a good balance of performance and efficient use of computational resources.

## 4. Mycobacterium tuberculosis in Kaohsiung, Taiwan

We apply the case-control approach for pairwise analysis to the full dataset which includes 4,154 individuals with Mtb in Kaohsiung, Taiwan. We choose five controls per case and note that the pairwise clustering proportion of the full dataset is 0.0006, including n = 5,453 1’s pairs and n = 27,265 0’s pairs formed from n = 4,154 individuals. We perform the analysis on the Bouchet HPC cluster with 200GB allocated memory and the full computation requires nearly 6 days (8,428.57 mins) to complete. The computation time of the full dataset without sampling is unknown, but we expect it to be substantially longer than this based on the results we show in Fig 7. We find that smaller age difference between the paired individuals (OR = 0.93 for every two-year increase in age difference, 95% CrI: 0.92-0.94), younger total age (OR = 0.87 for every two-year increase in the combined age, 95% CrI: 0.86-0.88), and closer geographic proximity (OR = 0.61 for every 5 km increase in spatial distance, 95% CrI: 0.57-0.65) were associated increased odds of being in the same genomic cluster.

To validate the results, we also apply the divide-and-conquer approach using the Meta method to combine posterior results across subsets for the whole dataset. We estimate standardized pairwise covariates from the full dataset and then randomly divided the full dataset into six subsets (700 cases for five subsets and 654 cases for one subset) to conduct pairwise analysis. The choice of subset size is based on our maximum available computational capacity. We ran these pairwise analyses on six subsets in parallel using the Bouchet HPC cluster with 50 GB of memory. The sequential computation time is 12,504.20 minutes, while the parallel computation time is 2,247 minutes, determined by the longest-running subset. Using this approach, we find that smaller age difference between the paired individuals (OR = 0.94 for every two-year increase in age difference, 95% CrI: 0.93-0.96), younger age (OR = 0.90 for every two-year increase in the combined age, 95% CrI: 0.88-0.91), and closer geographic proximity (OR = 0.73 for every 5 km increase in spatial distance, 95% CrI: 0.69-0.77) were associated increased odds of being in the same genomic cluster.

We compared results from two strategies in Fig 8. Overall, the results showed overall similar patterns. The posterior estimates of two age-related non-spatial factors were closely aligned, whereas the estimate of spatial distance showed a larger discrepancy. Such differences were consistent with our simulation results. For both the case-control and divide-and-conquer analyses, we collected 5,000 posterior samples after discarding the first 10,000 iterations as burn-in and thinning the remaining iterations by a factor of 5 to reduce autocorrelation. Using the same MCMC sampling parameters, we assessed convergence and found that the case-control approach exhibited better mixing and convergence than the divide-and-conquer approach (see S10 Fig). Given the poorer bias performance observed in the simulation study and the poorer convergence, the case-control approach provides more reliable estimates.

**Fig 8:**
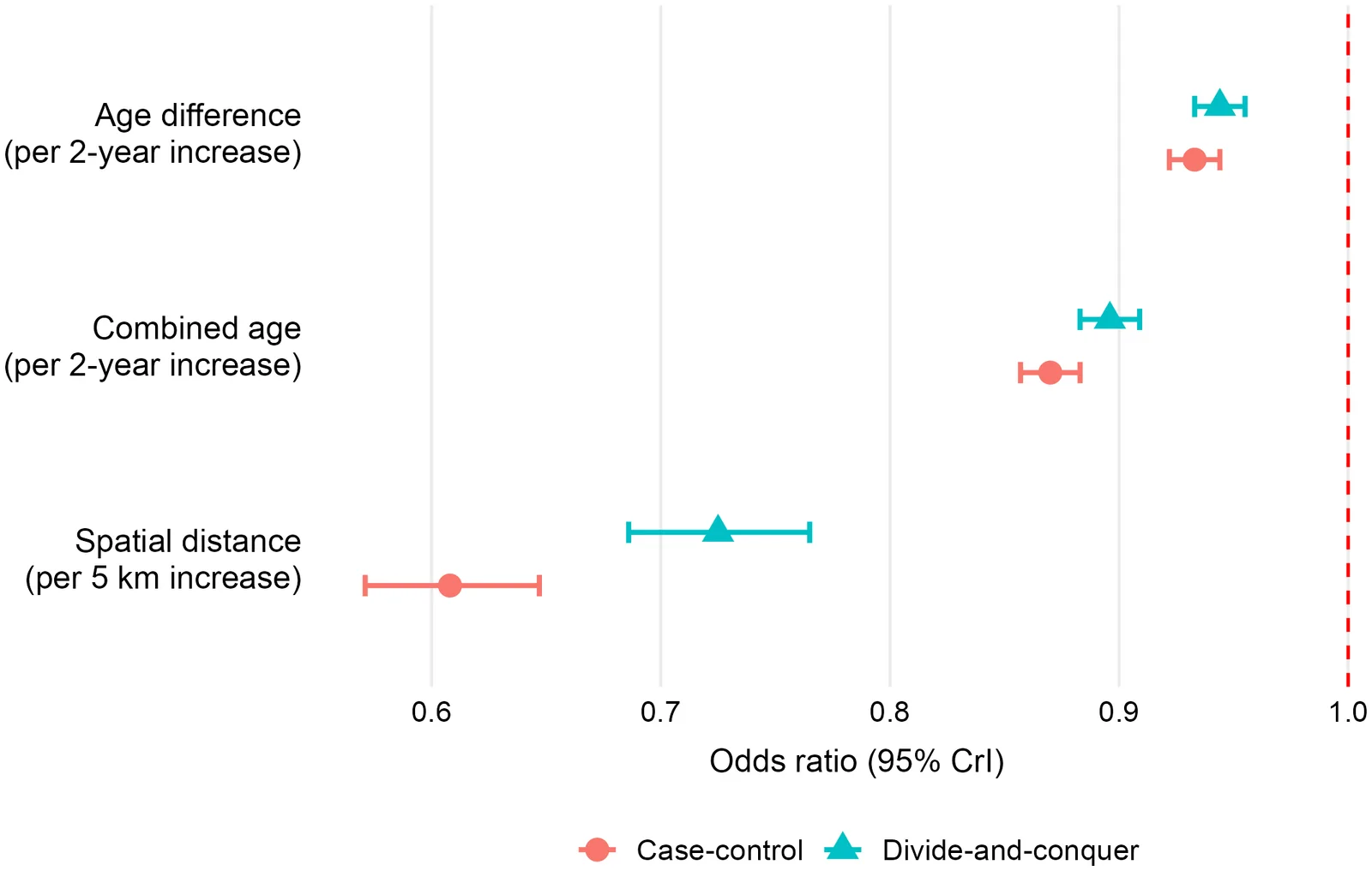
Comparison of odds ratio posterior median and 95% credible intervals for predictors on the full dataset using the case-control and divide-and-conquer methods.

## 5. Conclusion and discussion

In this study, we compare case-control and divide-and-conquer strategies to address computational challenges for pairwise analysis of genomic and spatial data in a spatial Bayesian setting. We evaluate these methods in term of the bias, coverage and length of the credible intervals, and computation time. Our results suggest that a 1:5 control-to-case sampling ratio provides a practical balance between estimation accuracy and computational efficiency for datasets with clustering characteristics similar to those represented in our simulation study.

We also observe that the case-control approach performs better than divide-and-conquer for estimates related to the spatial covariate, while both approaches produced similar non-spatial estimates (e.g., age difference) across subdivision levels (Figs 4–6). As we increased the number of subdivisions, the spatial distance estimates performed worse, with wider bias distributions and lower coverage, and the spatial factor also exhibits a consistently negative bias across subdivision levels. We find that this issue is driven by the large effect size rather than the spatial nature of covariate (S4 Fig). Accordingly, we suggest the case-control method is preferred as it appears more robust to the effect sizes associated with covariates of interest.

One possible explanation for the poorer performance of the divide-and-conquer approach is that partitioning the dataset across subsets effectively removes dyads spanning different subsets from the analysis. Because clustered dyads are extremely sparse, loss of these informative positive pairs may disproportionately weaken the transmission signal and contribute to attenuation of effect estimates toward the null. In contrast, the case-control approach retains all clustered dyads while subsampling only non-clustered pairs, thereby preserving the primary informative signal underlying the exposure–outcome relationship. This may explain its improved precision and reduced bias relative to the divide-and-conquer approach.

Although the case-control approach substantially reduces the computational burden compared with analyzing all pairwise observations, the runtime may still be considerable for very large datasets. In addition, increasing the control-to-case sampling ratio increases the computational cost, creating a trade-off between computational efficiency and the amount of information included in the analysis.

Our study has several limitations. First, because the true parameter values are unknown, we used parameter estimates from the three benchmark datasets as the reference values for generating simulated datasets and comparing the two approaches. Consequently, if the parameter estimates from the benchmark datasets are themselves biased, the simulated datasets and the resulting performance assessments may also inherit this bias. Second, although we considered three representative clustering proportions based on published TB genomic studies, the simulated datasets may not capture the full range of epidemiological scenarios encountered in practice. For example, studies focusing exclusively on multidrug-resistant TB may exhibit substantially higher clustering proportions than those considered here. Another limitation is that our simulations did not explicitly evaluate alternative transmission network structures (e.g., sparse versus dense networks), varying covariate effect sizes, or different sample sizes. Future studies should investigate the performance of the proposed methods under these additional epidemiological settings to further assess their robustness and generalizability. In addition, only random partitioning was considered for the divide-and-conquer approach. Because the true transmission network is generally unknown in practice, identifying an optimal partitioning strategy remains an important topic for future research.

Future work should investigate alternative analytic approaches that can balance pair retention, computational efficiency, and bias reduction across a wider range of data structures and outcome types. The outcome of the model we investigated in this study is binary, while other pairwise outcomes such as SNP distance, patristic distance, or probability of transmission may also be of interest. In these cases, the issue of loss pairs after subsampling are not relevant, but other challenges are introduced–––such as the selection criteria for pairs with a large genetic distance (i.e., those not possibly associated with transmission) when subsampling the full dataset.

Pairwise analysis plays a critical role in understanding transmission, as it can uncover associations between person-to-person transmission. Bayesian network and spatial methods represent powerful tools in this setting but are computationally demanding. Our study identifies computationally tractable alternatives to a full dataset analysis that can preserve inference for the primary regression parameters of interest. Building on our approach to large paired genomic and spatial data, future studies should explore novel ways of pairwise analysis on TB and other infectious diseases, including pairwise pathogen, individual, and environmental risk factors.

## Supporting information

S1 Table Clustering details from recent TB studies. (XLSX)

S1 Text Details of the literature search on pairwise clustering proportion. (DOCX)

S1 Fig Pairwise clustering proportions from recent published whole-genome sequencing TB studies. (TIFF)

S2 Fig MCMC trace plots for the case-control (1:1) design under clustering proportions of 0.008, 0.002, and 0.0009. (TIFF)

S3 Fig MCMC trace plots for the divide-and-conquer (5 subsets) design under clustering proportions of 0.008, 0.002, and 0.0009. (TIFF)

S4 Fig MCMC trace plots for the divide-and-conquer (5 subsets) design under clustering proportions of 0.008, 0.002, and 0.0009. (TIFF)

S5 Fig Mean 95% credible interval width and the corresponding 95% confidence intervals by method and factor using the case-control approach. (TIFF)

S6 Fig Estimated bias and empirical 95% credible interval coverage simulation study results by factors and methods using the divide-and-conquer approach. (TIFF)

S7 Fig Mean 95% credible interval width and the corresponding 95% confidence intervals for the mean by method and factors using the divide-and-conquer approach. (TIFF)

S8 Fig Estimated bias and empirical 95% credible interval coverage simulation study results (0.0009) by factors and methods after swapping the effect sizes of age difference and spatial distance using the divide-and-conquer approach. (TIFF)

S9 Fig Comparison of mean 95% credible interval width and the corresponding 95% confidence intervals between the case-control approach with five control per case and the divide-and-conquer approach with three subsets using the Meta method to combine. (TIFF)

S10 Fig Trace plots for the case-control and divide-and-conquer approaches applied to the full dataset using the same MCMC sampling procedure. (TIFF)

## References

[1] Bryant JM, Schürch AC, van Deutekom H, Harris SR, de Beer JL, de Jager V, et al. Inferring patient to patient transmission of Mycobacterium tuberculosisfrom whole genome sequencing data. BMC Infect Dis. 2013;13(1). doi: 10.1186/1471-2334-13-110 PMC3599118 23446317

[2] Stimson J, Gardy J, Mathema B, Crudu V, Cohen T, Colijn C. Beyond the SNP threshold: Identifying outbreak clusters using inferred transmissions. Mol Bio Evol. 2019;36(3):587–603. doi: 10.1093/molbev/msy242 PMC6389316 30690464

[3] Didelot X, Kendall M, Xu Y, White PJ, McCarthy N. Genomic epidemiology analysis of infectious disease outbreaks using transphylo. Curr Protocols. 2021;1(2). doi: 10.1002/cpz1.60 PMC7995038 33617114

[4] Campbell F, Didelot X, Fitzjohn R, Ferguson N, Cori A, Jombart T. outbreaker2: A modular platform for outbreak reconstruction. BMC Bioinformatics. 2018;19(S11). doi: 10.1186/s12859-018-2330-z PMC6196407 30343663

[5] Lan Y, Rancu I, Chitwood MH, Sobkowiak B, Nyhan K, Lin H-H, et al. Integrating genomic and spatial analyses to describe tuberculosis transmission: A scoping review. The Lancet Microbe. 2025;6(8):101094. doi: 10.1016/j.lanmic.2025.101094 PMC12371702 40228509

[6] Warren JL, Chitwood MH, Sobkowiak B, Colijn C, Cohen T. Spatial modeling of mycobacterium tuberculosis transmission with dyadic genetic relatedness data. Biometrics. 2023;79(4):3650–63. doi: 10.1111/biom.13836 PMC10404301 36745619

[7] Fithian W, Hastie T. Local case-control sampling: Efficient subsampling in imbalanced data sets. Ann Statist. 2014;42(5). doi: 10.1214/14-aos1220 PMC4258397 25492979

[8] Breslow NE. Statistics in epidemiology: The case-control study. J Am Stat Assoc. 1996;91(433):14–28. doi: 10.1080/01621459.1996.10476660 12155399

[9] Wang L, Williams ML, Chen Y, Chen J. Novel two‐phase sampling designs for studying binary outcomes. Biometrics. 2019;76(1):210–23. doi: 10.1111/biom.13140 PMC7042058 31449330

[10] Wang H, Zhu R, Ma P. Optimal subsampling for large sample logistic regression. J Am Stat Assoc. 2018;113(522):829–44. doi: 10.1080/01621459.2017.1292914 PMC6075720 30078922

[11] Wang J, Zou J, Wang H. Sampling with replacement vs poisson sampling: a comparative study in optimal subsampling. IEEE Trans Inform Theory. 2022;68(10):6605–30. doi: 10.1109/tit.2022.3176955

[12] Wang C, Srivastava S. Divide-and-conquer Bayesian inference in hidden Markov models. Electron J Statist. 2023;17(1). doi: 10.1214/23-ejs2118

[13] Guhaniyogi R, Banerjee S. Meta-kriging: Scalable bayesian modeling and inference for massive spatial datasets. Technometrics. 2018;60(4):430–44. doi: 10.1080/00401706.2018.1437474 PMC6474670 31007296

[14] Orozco-Acosta E, Adin A, Ugarte MD. Scalable Bayesian modelling for smoothing disease risks in large spatial data sets using INLA. Spatial Statistics. 2021;41:100496. doi: 10.1016/j.spasta.2021.100496

[15] Scott SL, Blocker AW, Bonassi FV, Chipman HA, George EI, McCulloch RE. Bayes and big data: the consensus Monte Carlo algorithm. Big Data and Information Theory. Routledge. 2022. p. 8–18. doi: 10.4324/9781003289173-2

[16] Neiswanger W, Wang C, Xing E. Asymptotically exact, embarrassingly parallel MCMC. 2013. https://arxiv.org/abs/1311.4780

[17] Xu J, Hu H, Ellison G, Yu L, Whalen CC, Liu L. Bayesian estimation of transmission networks for infectious diseases. J Math Biol. 2025;90(3). doi: 10.1007/s00285-025-02193-1 PMC13356605 39934500

[18] Li X, Chadwick F, Swallow B. Advances in approximate Bayesian inference for models in epidemiology. Epidemics. 2025;53:100855. doi: 10.1016/j.epidem.2025.100855 40992245

[19] O’Neill PD. A tutorial introduction to Bayesian inference for stochastic epidemic models using Markov chain Monte Carlo methods. Math Biosci. 2002;180(1–2):103–14. 10.1016/s0025-5564(02)00109-8 12387918

[20] Wu CY, Chen YA, Ioerger TR, Lan Y, Bai RY, Li MH. Lineage-specific transmission and spatial clustering of Mycobacterium tuberculosis in Kaohsiung, Taiwan, in 2019–23: a population-based genomic study. The Lancet Microbe. 2026. 10.1016/j.lanmic.2026.101369 42392122

[21] Silverman BW. Density estimation for statistics and data analysis. Routledge. 2018.

[22] Viechtbauer W. Conducting meta-analyses in R with the metafor package. J Stat Soft. 2010;36:1–48.

[23] Miroshnikov A, Conlon EM. parallelMCMCcombine: An R package for bayesian methods for big data and analytics. PLoS ONE. 2014;9(9):e108425. doi: 10.1371/journal.pone.0108425 PMC4178156 25259608

