# Supplementary material for: Comparing subsampling strategies for efficient pairwise analysis of large pathogen genomic and spatial datasets: An application to Mycobacterium tuberculosis transmission
Source: PLoS Comput Biol. 2026 Sep 23;22(9):e1014353. doi: 10.1371/journal.pcbi.1014353 (PMC13626476; doi:10.1371/journal.pcbi.1014353)
Supplement: Supplementary file 1 [file pcbi.1014353.s002.docx]

**Supporting information**

**Comparing subsampling strategies for efficient pairwise analysis of large pathogen genomic and spatial datasets: an application to *Mycobacterium* *tuberculosis* transmission**

Yu Lan^1^, Chieh-Yin Wu^2^, Hsien-Ho Lin^2^, Ted Cohen^1*^, Joshua L. Warren^3*^

^1^Department of Epidemiology of Microbial Diseases, Yale School of Public Health, New Haven, CT, USA

^2^College of Public Health, National Taiwan University, Taipei, Taiwan

^3^Department of Biostatistics, Yale School of Public Health, New Haven, CT, USA

^*^ Co-senior author

**Literature search on pairwise clustering proportions.** We searched recently published tuberculosis genomic epidemiology studies that reported the number and size of genomic clusters to identify representative clustering proportions for this study. We identified 14 studies published between 2014 and 2026 that provided sufficient clustering information to calculate the pairwise clustering proportion (Table S1 and Figure S1). Based on the distribution of these values, we selected three representative pairwise clustering proportions (0.0009, 0.002, and 0.008) to represent low, median, and high clustering settings in our simulation study.

We excluded three studies with substantially higher pairwise clustering proportions than the remaining studies. For example, Alvarez et al. (2021) reported that all cases (n=140) belonged to genomic clusters, resulting in a pairwise clustering proportion of 0.44. Although evaluating method performance under such highly clustered settings would be of interest, these studies were not considered representative of the broader range of clustering proportions reported in the tuberculosis genomic epidemiology literature. Investigation of such extreme scenarios is left for future work.

**References**

1. **Smith JP, Cohen T, Dowdy DW, Shrestha S, Gandhi NR, Hill AN.** Quantifying Mycobacterium tuberculosis transmission dynamics across global settings: a systematic analysis. ***American Journal of Epidemiology.*** 2023;192(1):133–145. doi:10.1093/aje/kwac149.
2. Alvarez GG, Zwerling AA, Duncan C, et al. Molecular epidemiology of *Mycobacterium tuberculosis* to describe the transmission dynamics among Inuit residing in Iqaluit, Nunavut using whole-genome sequencing. *Clinical Infectious Diseases*. 2021;72(12):2187–2195.
3. Asare P, Otchere ID, Bedeley E, et al. Whole genome sequencing and spatial analysis identifies recent tuberculosis transmission hotspots in Ghana. *Frontiers in Medicine*. 2020;7:161.
4. Guerra-Assunção JA, Crampin AC, Houben RMGJ, et al. Large-scale whole genome sequencing of *Mycobacterium tuberculosis* provides insights into transmission in a high prevalence area. *eLife*. 2015;4.
5. Verza M, Scheffer MC, Salvato RS, et al. Genomic epidemiology of *Mycobacterium tuberculosis* in Santa Catarina, southern Brazil. *Scientific Reports*. 2020;10:12891.
6. Walker TM, Lalor MK, Broda A, et al. Assessment of *Mycobacterium tuberculosis* transmission in Oxfordshire, UK, 2007–12, with whole pathogen genome sequences: an observational study. *The Lancet Respiratory Medicine*. 2014;2(4):285–292.
7. Chee CBE, Lim LKY, Ong RTH, et al. Whole genome sequencing analysis of multidrug-resistant tuberculosis in Singapore, 2006–2018. *European Journal of Clinical Microbiology & Infectious Diseases*. 2021;40(5):1079–1083.
8. Macedo R, Pinto M, Borges V, et al. Evaluation of a gene-by-gene approach for prospective whole-genome sequencing-based surveillance of multidrug resistant *Mycobacterium tuberculosis*. *Tuberculosis*. 2019;115:81–88.
9. Jiang Q, Liu Q, Ji L, et al. Citywide transmission of multidrug-resistant tuberculosis under China's rapid urbanization: a retrospective population-based genomic spatial epidemiological study. *Clinical Infectious Diseases*. 2019;71(1):142–151.
10. Yang C, Luo T, Shen X, et al. Transmission of multidrug-resistant *Mycobacterium tuberculosis* in Shanghai, China: a retrospective observational study using whole-genome sequencing and epidemiological investigation. *The Lancet Infectious Diseases*. 2017;17(3):275–284.
11. Meumann EM, Horan K, Ralph AP, et al. Tuberculosis in Australia's tropical north: a population-based genomic epidemiological study. *The Lancet Regional Health – Western Pacific*. 2021;15:100229.
12. Rodríguez-Grande C, Vallejo-Godoy S, Martínez-Lirola M, et al. Long-term refined genomic analysis of tuberculosis clusters to distinguish between ongoing transmission, reactivations or diagnostic delays, Almería, Spain, 2003 to 2024. *Eurosurveillance*. 2026;31(11):2500301.
13. Zhang X, Martinez E, Lam C, et al. Exploring programmatic indicators of tuberculosis control that incorporate routine *Mycobacterium tuberculosis* sequencing in low incidence settings: a comprehensive (2017–2021) patient cohort analysis. *The Lancet Regional Health – Western Pacific*. 2023;41.
14. Liu D, Huang F, Li Y, et al. Transmission characteristics in tuberculosis by whole-genome sequencing: nationwide cross-sectional surveillance in China. *Emerging Microbes & Infections*. 2024;13(1):2348505.
15. Lan Y, Crudu V, Ciobanu N, et al. Identifying local foci of tuberculosis transmission in Moldova using a spatial multinomial logistic regression model. *EBioMedicine*. 2024;102.
